# Implementation of a preoperative fasting abbreviation protocol in a tertiary pediatric center

**DOI:** 10.6061/clinics/2021/e2995

**Published:** 2021-07-26

**Authors:** Adriana S. Gandolfo, Priscilla F.N. Cardoso, Izabel M. Buscatti, Manoel Carlos P. Velhote, Maria Aparecida C. Bonfim, Alberto C. Helito

**Affiliations:** Instituto da Crianca e do Adolescente, Hospital das Clinicas HCFMUSP, Faculdade de Medicina, Universidade de Sao Paulo, Sao Paulo, SP, BR.

Over the past two decades, the concept of preoperative fasting for pediatric patients undergoing elective surgery has been reviewed by the scientific medical community. The European Society of Anesthesiology and American Society of Anesthesiology announced a minimum of 2h of preoperative fasting time for children and adolescents and the ingestion of only clear fluids within 2h prior to procedural anesthesia (1,2).

We currently know that more than 4h of fasting may lead to unfavorable metabolic and inflammatory effects, such as increased insulin resistance, hunger, and thirst, in the pediatric population and can negatively impact a child’s mental well-being. These aspects result in worse response to surgical trauma, which may lead to negative outcomes in terms of postoperative morbidity, length of hospitalization, and complications (2).

A prospective, multicenter, randomized study that evaluated 1200 children found that blood glucose levels at the time of anesthesia were significantly higher in all groups that received up to 15 ml/kg of a 10% carbohydrate solution 2h before anesthesia compared with the 6 h preoperative fasting group (control group). Other relevant findings included no residual gastric volume in the groups receiving oral glucose administration up to 10 ml/kg and a significantly higher crying ratio in the control group than in the other three groups (3). Aguilar-Nascimento et al. reported better metabolic and inflammatory responses by reducing the preoperative fasting time in preschool children up to 2h (4).

Furthermore, in a multicenter study that enrolled over 30,000 children in Europe, pulmonary aspiration was observed in only 0.1% of the patients and was not associated with an increase in morbidity and mortality in this population (5).

Recently, a new proposal advocating the reduction of fasting time for clear fluids from 2h to 1h was introduced (6,7). Previous studies have shown promising results in shortening the clear fluid-fasting regimen in children. Indeed, there was no increase in the incidence of bronchial aspiration and no difference between gastric residual volumes when the clear fluid fasting time was 1h or 2h. Moreover, changing the fasting period from 2h to 1h reduced the median clear fluid fasting period from 4h to 1h and raised the number of children subjected to fasting times less than 4h by up to 72% (8-13).

At our institution, an analysis of fasting time indicators showed that almost half of the day hospital surgical patients had a fasting time longer than 12h. Apprehension towards surgery suspension by caregivers and the fear of clinical complications by health teams may be potential reasons for this finding. Based on this, we proposed the adoption of an institutional protocol for preoperative fasting abbreviations. The main objective of the protocol was awareness among health teams, patients, and caregivers about the safety and benefits of shortening the fasting period that children and adolescents were subjected to before elective surgery.

A committee of professionals was created, including medical teams, nurses, and nutritionists from the Pediatric Day Hospital Unit, Pediatric Surgery Unit, and Anesthesiology Unit. The protocol was as follows: **1.** clear liquids were prescribed in the hospital up to 2h before elective surgery, with respect to the exclusion criteria (Table 1); **2.** caregivers were provided an orientation about the possibility of offering different kinds of food on the same day of the procedure, respecting safe intervals, such as light meals up to 8h prior to hospital arrival, formula up to 6h prior to hospital arrival, breast milk up to 4h prior to hospital arrival, and clear liquids (gelatin and coconut water) up to 2h prior to hospital arrival; and **3.** sending text messages for guidance, the day before the procedure. Since 2016, the committee has periodically audited the protocol based on current indicators and recent literature.

The protocol was implemented in July 2016. The first departments that adopted the protocol were the Pediatric Day Hospital Unit and Pediatric Surgery Unit. Then, it was adopted by all units of the Children and Adolescents’ Institute, Hospital das Clinicas, Faculdade de Medicina, Universidade de São Paulo (HCFMUSP), and by other institutes in our tertiary hospital complex. After protocol implementation, there was a statistically significant reduction in preoperative fasting time: >6h (256 [94.1%] patients before protocol *versus* 473 [60.8%] after protocol); >10h (194 [71.3%] patients before protocol *versus* 233 [29.9%] after protocol); and >12h (129 [47.4%] patients before protocol *versus* 111 [14.3%] after protocol) (*p*<0.001 for all), without the occurrence of adverse events, such as bronchopulmonary aspiration with clinical repercussion and/or related morbidities or even suspension of surgeries. Indeed, at the Pediatric Day Hospital Unit, there were fewer episodes of symptomatic hypoglycemia, dehydration, and the need for a venous line for treatment. The standard procedure for general anesthesia in children in our hospital is inhalation induction. Venous puncture prior to operatory room arrival is unusual and may cause discomfort to patients or caregivers. Table 2 compares the fasting time and venous puncture indicators before and after protocol implementation.

The presented data confirm the importance and beneficial impact of the fasting time abbreviation protocol in pediatric healthcare. As a result of the shorted fasting periods, feelings of anxiety, fear, pain, sadness, hunger, thirst, discomfort, and malaise were minimized. They also favor faster post-surgical recuperation.

As the protocol continues to evolve, the committee continuously seeks areas for improvement. Our next step would be shortening the clear liquid fasting period to 1h, in accordance with the latest scientific trends (6,7,13).

Thus, this protocol provides better patient outcomes and the opportunity for shared decision-making with patients and caregivers at our health facility.

## Figures and Tables

**Table 1:** Exclusion criteria for clear liquid prescription after hospital admission.

Patients undergoing pharmacological treatment for gastroesophageal reflux disease (or previous treatment in the last 12 months)
Patients using drugs that delay gastric emptying (cyclosporine, opioids, amitriptyline, calcium channel blockers, octreotide, chlorpromazine, and promethazine)
Patients with neurological diseases
Patients diagnosed with peritonitis, intestinal obstruction, diabetes, severe obesity, significant gastrointestinal bleeding, or pregnancy
Patients in maintenance fluid therapy or parenteral nutrition
Patients on peritoneal dialysis or chronic hemodialysis
Patients preparing for a positron emission tomography and computed tomography scan

**Table 2:** Demographic data, preoperative fasting time, and the need for a venous line before elective surgery for Pediatric Day Hospital Unit patients.

Variables, n=1050	Before protocol (n=272)	After protocol (n=778)	*p*-value
Demographic data			
Age, months	46.8 (0-235)	58.5 (1-273)	0.027
Male sex	201 (73.9)	550 (70.7)	0.352
Comorbidities	101 (37.1)	357 (45.9)	0.015
Preoperative fasting time			
>6 hours	256 (94.1)	473 (60.8)	<0.001
>10 hours	194 (71.3)	233 (29.9)	<0.001
>12 hours	129 (47.4)	111 (14.3)	<0.001
Venous punction	41 (15.1)	13 (1.7)	<0.001

Results are presented as median (minimum value-maximum value) or numbers (%). Data were compared using a Mann-Whitney test and chi-squared test.
